# Biomimetic Design of Dental Restorative Materials: Conceptual Framework and Translational Challenges

**DOI:** 10.3390/biomimetics11040256

**Published:** 2026-04-07

**Authors:** Tasneem Alluhaidan, Benjamin Hung, Masoumah Qaw, Isadora M. Garcia, Mary Anne S. Melo

**Affiliations:** 1 Dental Biomedical Sciences Ph.D. Program, University of Maryland School of Dentistry, Baltimore, MD 21201, USA; talluhaidan@umaryland.edu (T.A.); mqaw@umaryland.edu (M.Q.); 2 Department of Preventive Dental Sciences, College of Dentistry, Imam Abdulrahman Bin Faisal University, Dammam 31441, Saudi Arabia; 3 Department of Comprehensive Dentistry, University of Maryland School of Dentistry, Baltimore, MD 21201, USAigarcia1@umaryland.edu (I.M.G.); 4 Department of Restorative Dental Sciences, College of Dentistry, Imam Abdulrahman Bin Faisal University, Dammam 31441, Saudi Arabia

**Keywords:** biomimetic, bioactive, bioinspired, dental composites, dental materials, antibacterial, ion-releasing

## Abstract

Biomimetics in dental restorative materials has gradually shifted from simply copying the appearance of natural teeth to better understanding how those tissues actually behave. Instead of focusing only on aesthetics, there is now more attention on how enamel and dentin function in real conditions, how they respond to stress, interact with their surroundings, and change over time. Because of this, newer materials are no longer just passive fillers; they are being designed to reflect aspects of natural tooth structure, composition, and behavior within the oral environment. This review brings together key ideas in this area, recent developments, and the challenges that remain. One issue that often comes up is how terms like bioinspired, biomimetic, and bioactive are used. They are sometimes treated as if they mean the same thing, but in practice, they point to different goals or levels of complexity in material design. For instance, some studies focus on creating more organized composite structures or mimicking natural mineralization processes, while others focus on antibacterial surfaces or peptide-based approaches that may support remineralization. There is also growing interest in materials that respond to environmental changes, such as shifts in pH or the early stages of wear. Even with promising laboratory results, these materials are not yet widely used in everyday clinical practice. Several issues continue to slow their adoption, including unclear terminology, limited availability of testing models that reflect real oral conditions, and a lack of long-term clinical data. Part of the challenge lies in the lack of consistent terminology, which can make it harder to compare findings across studies. Manufacturing challenges also remain, particularly when scaling up more complex systems. Moving forward, progress will depend on closer collaboration across disciplines, including materials science, oral biology, microbiology, and digital manufacturing. Such efforts will be important for developing restorative materials that behave more like natural tissues and perform reliably over time inside the mouth.

## 1. Introduction

Dental restorative materials have traditionally been developed to restore the form and mechanical integrity of damaged tooth tissues [1]. Despite major advances, conventional approaches remain limited in their ability to reproduce the biological complexity, structural hierarchy, and dynamic behavior of natural dentition [2,3]. Teeth are not passive structures but living, multifunctional systems composed of highly organized tissues that provide mechanical resilience while supporting continuous mineral exchange, microbial defense, and interaction with saliva and surrounding tissues [4,5].

The growing interest in biomimetics reflects a broader shift in restorative materials science toward understanding how natural mineralized tissues are formed and function [6,7]. In biological systems, enamel, dentin, bone, and other hard tissues arise through biomineralization processes in which organic matrices guide the nucleation, growth, and hierarchical assembly of inorganic crystals across multiple length scales [8]. These tightly regulated interactions produce materials that combine high mechanical strength with dynamic biological functionality, properties that conventional synthetic restoratives have not been able to replicate [9].

Biomimetic strategies in dentistry therefore seek to move beyond filling the defect toward reproducing the underlying design principles of natural tissues [10]. This includes mimicking hierarchical organization, template-directed mineral growth, ion-mediated repair mechanisms, and the integration of organic and inorganic phases that allow natural tissues to resist damage while maintaining biological responsiveness [11].

In this context, biomimetic restorative materials are increasingly viewed as approaches that aim to emulate nature’s design logic, recreating the stress distribution, adaptive repair, and tissue integration observed in enamel and dentin [12,13]. Within this emerging field, three often-overlapping terms; bioinspired, biomimetic, and bioactive, describe different levels of engagement with natural principles. Each plays a distinct role in the conceptualization and development of modern restorative materials (Box 1). These categories describe different levels of interaction with biology, rather than separate material types, which is why they can overlap. A material can be biomimetic because it reproduces the structure or functional behavior of natural tissues, and at the same time be bioactive if it also influences a specific biological process.

Box 1 **Core Definition for Bioinspired, Biomimetic, and Bioactive Dental Materials.**

*Bioinspired materials*: designed using concepts derived from natural structures or processes, without replicating biological mechanisms or tissue organization. Their relationship to biology is analogical rather than functional.

*Biomimetic materials*: engineered to reproduce the structural hierarchy, physicochemical behavior, or functional mechanisms of natural tissues, often through controlled interactions between organic and inorganic phases.

*Bioactive materials*: intentionally and specifically modulate a targeted biological process to produce a beneficial therapeutic outcome. True bioactivity requires active biological interaction rather than passive chemical effects such as simple ion release or mineral precipitation.

The key distinction is that biomimicry concerns how closely a material imitates natural tissue design, whereas bioactivity concerns whether the material actively changes biological behavior. For example, a restorative material that recreates the hierarchical organization of dentin would be considered biomimetic. It would only qualify as bioactive if it also demonstrated a targeted biological effect, such as regulating mineralization pathways, modulating cellular signaling, or controlling cariogenic biofilm activity through defined mechanisms.

In other words, biomimetic materials may or may not be bioactive. Still, a material cannot be considered truly bioactive unless there is clear evidence that it intentionally and specifically alters a biological pathway to produce a beneficial therapeutic outcome.

This review examines how biomimetic principles are being applied in the design of restorative dental materials, from their conceptual foundations to more recent innovations and the challenges that still limit clinical translation. To clarify how terms such as bioinspired, biomimetic, and bioactive are used in this field, we outline a working conceptual framework and then explore how these ideas influence the development of restorative composites, adhesives, liners, and tooth–material interfaces.

The paper is based on a narrative review of the literature, drawing on publications indexed in databases such as PubMed, Scopus, and Web of Science. Searches were conducted using combinations of relevant keywords, and selected studies were chosen not only for their focus on restorative dental biomaterials but also for their contributions to shaping the conceptual perspective presented here.

## 2. Conceptual Framework

The terminology used to describe biologically driven dental materials reflects progressive levels of engagement with natural systems, rather than distinct or mutually exclusive material categories. As outlined in Box 2, bioinspired, biomimetic, and bioactive materials can be understood along a continuum defined by the depth of biological emulation and the nature of their interaction with living tissues.

Box 2 **Conceptual Framework for Classifying Biologically Driven Dental Materials.**

**I.**  **Structural and Design Relationship to Biology**

This category explains the extent to which a material reflects natural structural patterns or biological principles.
CriterionBioinspiredBiomimeticBioactiveUses natural design principlesYesYesNot requiredReplicates tissue structureNoYesNot requiredMimics biological mechanismsNoYesNot requiredRequires hierarchical organizationNoTypicallyNot required

**II.**  **Level of Biological Evidence**

This dimension describes increasing levels of biological engagement, ranging from no interaction to measurable therapeutic outcomes.
BioinspiredNo direct biological interaction is required.BiomimeticBiological interaction may occur, but it is not mandatory for classification.BioactiveActive engagement with tissues or the surrounding biological environment is required. These materials must influence a defined biological pathway and demonstrate a measurable therapeutic effect.

**III.**  **Translational stages**

Each classification level generally corresponds to a different stage of development.
CriterionBioinspiredBiomimeticBioactiveTypical translational stageConceptual design/engineering validationMechanistic validation/preclinical developmentFunctional validation/clinical translation

**IV.**  **Minimum evidence required to claim classification**

To justify classification, the following minimum evidence is expected:
BioinspiredClear demonstration that the material design is derived from a biological analogy.BiomimeticValidation that structural or functional features replicate biological counterparts.BioactiveEvidence showing targeted modulation of a biological pathway with demonstrable functional impact.

Within this review, the conceptual framework serves as an organizing structure that integrates definitions, scientific evidence, and translational considerations into a coherent system [14]. Conceptual frameworks function as the “connective tissue” of a study, linking core constructs, clarifying their relationships, and providing a guide for interpretation and analysis. In this context, the framework allows biologically driven materials to be situated within a unified model that connects terminology, biological mechanisms, and clinical relevance.

It is worth noting that the terms *bioinspired*, *biomimetic*, and *bioactive* are not always used in a consistent way across biomaterials and dental materials literature. In quite a few papers, the boundaries between them are a bit blurred, or they are used more loosely to describe materials that, in general, interact with biology or resemble natural tissues in some way [15].

Here, though, we are using these terms in a more structured way. This classification is intended to provide conceptual clarity rather than to redefine existing terminology. Basically, bioinspired refers to materials that draw on ideas or design cues from nature; biomimetic refers to those that more closely replicate biological structures or processes; and bioactive describes materials that engage with biological systems, such as through chemical interactions or signaling pathways [15].

At the most fundamental level, bioinspired materials adopt design concepts derived from nature without reproducing biological structure or function. Their relationship to biology is analogical, with a primary focus on improving mechanical or physicochemical performance. As a result, they typically represent early stages of innovation, where conceptual translation into biological function has not yet occurred.

Biomimetic materials occupy an intermediate position within this framework. These systems aim to replicate key features of natural tissues, such as hierarchical organization, gradient structures, or biologically regulated mineralization processes. Their defining characteristic is the attempt to emulate how tissues function, rather than simply how they appear. Importantly, this materials-based definition should not be confused with “Biomimetic Dentistry” as a clinical philosophy [16], which refers to minimally invasive restorative techniques that preserve tooth structure and replicate natural biomechanics at the macroscopic level. In the present framework, biomimetic refers specifically to material design and the replication of biological mechanisms at the micro- and nano-scale, not to operative strategies or adhesive protocols.

At the highest level of biological integration are bioactive materials, defined not by passive interactions, but by their intentional capacity to modulate biological activity at the interface with host tissues. In this sense, bioactivity reflects a material’s ability to direct specific cellular and molecular responses through controlled interactions within the local microenvironment, rather than through incidental effects alone, such as ion release or surface reactivity [17].

Within this framework, the defining feature of bioactive restorative systems is their capacity to engage biological pathways that are relevant to tissue function, repair, or disease control. Recent developments in restorative materials increasingly incorporate biologically instructive components designed to target these pathways. For example, peptide-guided remineralization approaches use analogs of dentin matrix proteins, such as amelogenin or dentin phosphoprotein, to regulate mineral nucleation and promote intrafibrillar hydroxyapatite formation within collagen, enabling reconstruction of dentin architecture in a biomimetic way [6,18].

Other systems have been designed to deliver signaling cues that mimic endogenous regulatory molecules. Materials capable of releasing growth-factor-like signals, including BMP analogs, have shown the ability to drive dental pulp stem cells toward odontoblast-like differentiation and support reparative dentin formation. In parallel, enzyme-responsive materials have been developed to interact with matrix-degrading enzymes such as matrix metalloproteinases and cathepsins, thereby preserving the hybrid layer, stabilizing the adhesive interface, and supporting tissue repair processes [19]. Furthermore, enzyme-responsive materials have been engineered to modulate proteolytic enzymes associated with matrix turnover, including matrix metalloproteinases and cathepsins, thereby maintaining the hybrid layer and facilitating dentin regeneration [20].

Taken together, these strategies illustrate a shift from materials that merely interact with tissues to those deliberately engineered to guide biological responses. In line with current understanding, true bioactivity requires this level of intentional and mechanistically grounded modulation of biological systems, rather than reliance on indirect or nonspecific chemical effects.

Importantly, these categories represent nested levels of biological complexity. A material may be both biomimetic and bioactive, but bioactivity inherently implies a higher threshold of evidence, including targeted biological effects and clinically relevant outcomes. This hierarchical conceptualization also parallels translational readiness.

## 3. Biomimetic Strategies

**Figure 1:**
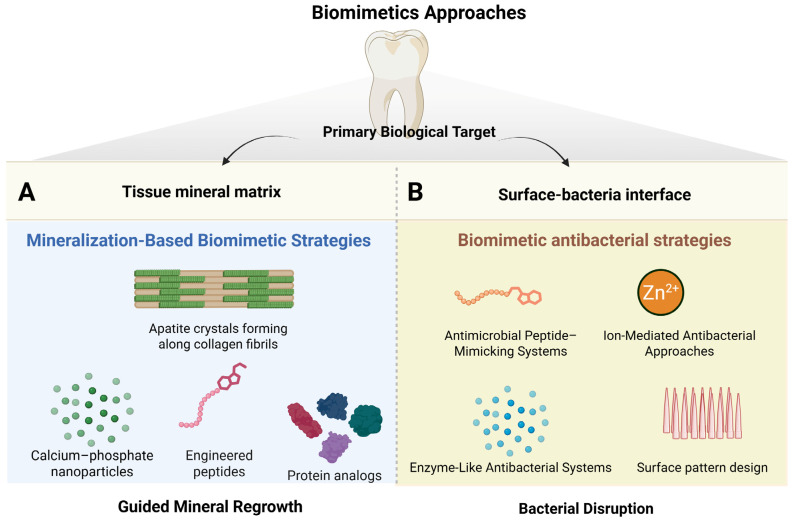
Overview of biomimetic strategies in restorative dentistry based on their primary biological target. Biomimetic approaches in dentistry can be broadly divided into two major categories. (**A**) Mineralization-based biomimetic strategies target the tissue mineral matrix and aim to restore lost tooth structure by guiding the formation of apatite crystals that resemble natural enamel or dentin biomineralization. These approaches commonly use calcium–phosphate systems, engineered peptides, protein analogs, and bioactive polymers to promote guided mineral regrowth along collagen fibrils. Some systems focus solely on mineral deposition, while others combine remineralization with antibacterial functionality. (**B**) Biomimetic antibacterial strategies primarily target the surface–bacteria interface and are designed to control microbial colonization rather than rebuild tissue. These include two mechanistic directions: (i) bioinspired surface architectures, such as nanotextured patterns modeled after natural antifouling surfaces that physically disrupt bacterial adhesion and early biofilm formation; and (ii) molecular biomimetic approaches that imitate host defense mechanisms, including antimicrobial peptide–mimicking systems, enzyme-like catalytic antibacterial systems that generate reactive oxygen species, and ion-mediated strategies that reproduce antimicrobial chemical environments using ions such as zinc or fluoride.

In dentistry, biomimetic strategies generally fall into two broad categories, as illustrated in Figure 1. One group focuses on mineralization, aiming to recreate enamel-like structures that support tissue integration and repair (Figure 1A) [21]. These approaches often rely on engineered peptides, protein analogs, bioactive polymers, or calcium phosphate systems that guide apatite formation [10,21]. Some are designed purely to promote mineral deposition [22] while others also exhibit antibacterial effects [23,24]. For instance, laser-assisted biomimetic methods can produce fluoride-containing apatite layers on dentin that bond closely to the substrate and suppress Streptococcus mutans [25]. Similarly, hybrid organomineral coatings combining polymerized hydroxyquinoline with nanocrystalline hydroxyapatite can reproduce enamel-like mechanical properties while reducing bacterial viability [23].

The second group centers on antibacterial strategies (Figure 1B), which can be broadly divided into two main biomimetic directions. One subset is based on bioinspired surface architecture, which is the primary focus of this section. These approaches draw on natural surfaces known to resist microbial colonization. Instead of reproducing biological chemistry or stimulating host responses, they mimic physical micro and nano-scale features that interfere with bacterial attachment and early biofilm formation. A classic example is nanotextured surfaces inspired by cicada wings, where tightly packed nanopillars can mechanically distort or rupture bacterial membranes upon contact. Because this effect occurs only at the material interface, these designs mainly influence early colonization [24].

Other biomimetic antibacterial strategies in dentistry follow a different path. Rather than copying surface structure, they imitate natural host defense mechanisms at the molecular level [26]. Many are inspired by antimicrobial peptides, reproducing their amphiphilic charge patterns to disrupt bacterial membranes selectively. Similar concepts are used in cationic polymers and ionic liquid-based systems that function through peptide-like contact killing [27,28]. Some approaches also mimic enzymatic defense pathways by incorporating catalytic components that generate reactive oxygen species locally, resembling oxidative antimicrobial responses in biological tissues [29]. In addition, ion-mediated systems attempt to recreate the antimicrobial chemical environment of mineralized tissues by incorporating ions such as zinc or fluoride into calcium phosphate phases [30].

Recognizing these differences is important. Bioinspired surface designs primarily act by controlling bacterial attachment at the interface, whereas molecular biomimetic approaches aim to reproduce biological antimicrobial functions.

### 3.1. Biomimetic Biomineralization

Biomimetic biomineralization in restorative dentistry centers on one simple idea: rather than simply restoring lost tooth structure with artificial materials, these approaches aim to help the tooth rebuild its own mineral in a way that resembles natural enamel or dentin formation [13]. This means copying, as closely as possible, the biological and chemical events that originally formed enamel and dentin. The field sits right at the crossroads of materials science, biology, and clinical dentistry, and over the past two decades, it has grown into one of the most active areas of translational dental research [9]. Most of the evidence so far comes from laboratory and preclinical work, with a smaller but growing body of early clinical studies [31]. Here, we are limiting the discussion to strategies relevant to caries prevention, early enamel repair, dentin stabilization, and restorative materials.

For simplicity, most biomimetic approaches rely on three basic components that work together. First, there is a guiding structure. In dentin, this is usually the existing collagen network. In engineered systems, it may be a scaffold made from peptides, recombinant proteins, or hydrogels [32]. This matrix acts as a framework that directs where and how minerals form. Its surface chemistry is designed to attract calcium and phosphate ions and organize them in a controlled way. Second, these systems use stabilized mineral building blocks. Instead of forming crystals immediately, calcium phosphate is kept in a temporary, amorphous state using polymers, casein-derived phosphopeptides, or similar molecules [33]. In this form, the mineral behaves almost like a fluid at the nano-scale, allowing it to enter tiny spaces, such as enamel pores or gaps within collagen fibrils, before hardening into apatite. Third, successful strategies depend on careful control of crystal formation. Factors such as pH, ion concentrations, and fluoride additives influence when and how the amorphous phase converts into organized apatite [34]. The goal is to produce a mineral that blends with, or closely resembles, natural enamel or dentin in structure and orientation.

At the enamel level, many newer systems build on a concept that has been around for years: delivering calcium and phosphate in a form that can actually penetrate early lesions before they crystallize. Amorphous calcium phosphate (ACP) remains central to this approach [35]. Recent studies show that when ACP is combined with biomimetic carriers, such as protein-like coatings or polysaccharide stabilizers, it can repair both surface and subsurface white spot lesions [36]. Some of these systems also add antibacterial effects, which is clinically important because remineralization without biofilm control has limited long-term value.

Another interesting direction tries to mimic what happens during natural enamel maturation. Experimental systems now use RNA-based stabilizers that temporarily hold calcium phosphate in an amorphous state [37]. As the RNA breaks down, crystal growth is triggered, producing denser, better-organized enamel-like mineral. From a clinical standpoint, this matters because one of the weaknesses of earlier remineralization technologies was that they produced soft or poorly structured mineral deposits.

There has also been progress with ion-modified calcium phosphate formulations. Products containing small amounts of fluoride, magnesium, carbonate, or strontium appear to encourage mineral growth that more closely resembles natural hydroxyapatite [37].

Now, dentin presents a different challenge because of its collagen matrix. The durability of resin–dentin bonding remains limited due to hydrolytic degradation and enzymatic breakdown of exposed collagen [38]. Newer biomimetic systems aim to deliver nano-scale mineral precursors that can infiltrate collagen fibrils and restore intrafibrillar mineral [39]. Adhesive systems have been used as the primary carrier of these systems, aiming to recapitulate the native biomineralization of dentin, transforming demineralized collagen into a mineralized hybrid interface [7]. Peptide-mediated nucleation systems, particularly those derived from amelogenin, dentin phosphoproteins, and statherin analogs, have been covalently tethered to adhesives to promote intrafibrillar mineral deposition and protect collagen matrices from enzymatic degradation [40]. For example, peptide-functionalized adhesives containing phosphorylated biomimetic analogs have accelerated in situ formation of carbonated hydroxyapatite within collagen fibrils, significantly increasing bond stability under cyclic fatigue [32,41]. Concurrently, enzyme-inhibiting biomimetic primers employing quaternary ammonium methacrylates or zinc-stabilized crosslinkers have demonstrated suppression of matrix metalloproteinases (MMPs) and cathepsins while preserving intertubular mineral content [42]. Others have evaluated chitosan-based gels containing nano-hydroxyapatite, Biosilicate, and L-aspartic acid for their ability to remineralize demineralized dentin, reinforce collagen, and modulate MMP activity [43].

These concepts also extend to restorative dentistry. Researchers have explored adhesives that slowly release calcium and phosphate while stabilizing collagen within the hybrid layer [44]. The results are promising but still experimental. Still, it is important to keep expectations realistic. Despite marketing claims, restorative materials primarily release ions for only a limited time, with minimal true apatite formation, though there are important nuances, and some materials do demonstrate genuine bioactive effects.

From a knowledge-translation perspective, most biomimetic mineralization strategies are still moving along the pathway from discovery to early invention. Laboratory studies clearly demonstrate feasibility, and some systems have progressed into prototype products and early clinical evaluation. However, only a limited number have reached widespread clinical adoption, reflecting the broader challenge of translating complex bioinspired material systems into durable, manufacturable, and regulatory-approved dental technologies.

### 3.2. Bioinspired and Biomimetic Antibacterial Strategies

**Figure 2:**
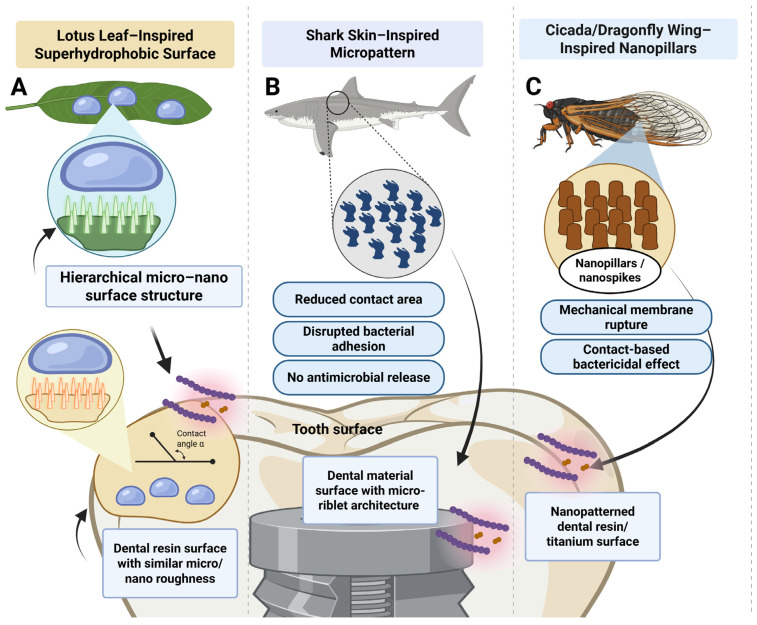
Representative examples of antibacterial surface topographies inspired by natural structures that resist microbial colonization through purely physical mechanisms: (**A**) Lotus leaf–inspired superhydrophobic surfaces rely on hierarchical micro- and nano-scale roughness combined with low surface energy to create high water contact angles. This architecture promotes a self-cleaning effect in which water droplets roll off the surface, carrying away debris and microorganisms. In dental materials, similar micro–nano textured resin surfaces can reduce protein adsorption and early bacterial attachment without releasing antimicrobial agents. (**B**) Shark skin–inspired micropatterns mimic the aligned riblet structures of dermal denticles that limit microbial accumulation. These microgrooved topographies reduce the effective contact area between bacteria and the material surface, disrupt stable adhesion, and interfere with early biofilm formation through physical rather than chemical effects. (**C**) Cicada and dragonfly wing–inspired nanopillars represent a contact-active bactericidal strategy based on dense nano-scale spikes that mechanically deform or rupture bacterial membranes upon contact. In dental applications, similar nanopatterned titanium and polymer surfaces demonstrate non-leaching, contact-based antibacterial activity against oral pathogens.

Bioinspired antibacterial surfaces mimic physical topographies found in nature that resist microbial attachment. Their primary mechanism is non-leaching, contact-based control of bacterial adhesion or viability, rather than chemical or biological modulation. The surfaces described in this section, inspired by lotus leaves, shark skin, and cicada wings, are categorized as ‘bioinspired’ according to the definition provided in Box 2. Bioinspired designs (Figure 2) in dental restorative materials harness structural features from nature, particularly those found in plants and animals that exhibit self-cleaning, antifouling, or antimicrobial properties, to guide synthetic material innovation [45]. Unlike biomimetic or bioactive strategies that often aim to replicate chemical or functional responses, bioinspired approaches focus on replicating physical architectures to disrupt microbial adhesion or biofilm formation [46].

From a translational perspective, these approaches are attractive because they are non-leaching and may reduce concerns related to the exhaustion of the releasing agent, toxicity, or the development of bacterial resistance. However, challenges remain in maintaining structural fidelity during manufacturing and ensuring stability under clinical conditions.

#### 3.2.1. Lotus Leaf-Inspired Superhydrophobic Surfaces

**Mechanistic basis:** The lotus leaf exhibits a classic “self-cleaning” or “superhydrophobic” effect due to its hierarchical micro–nano structures combined with a low surface energy wax coating [47]. This leads to extremely high water contact angles (>150°), which allow water droplets to roll off the surface, carrying debris and microbes away [48]. This phenomenon has inspired the development of superhydrophobic resin surfaces that repel protein adhesion and bacterial colonization [49]. Other hierarchical surface patterns inspired by rose petals clearly demonstrate the ability to reduce early bacterial attachment through purely physical mechanisms. These surfaces combine microscale papillae with nano-scale folds, creating a textured architecture that limits how bacterial cells can settle and make stable contact. Evidence from in vitro experiments demonstrates that this design can reduce initial attachment of common biofilm-forming species, such as Staphylococcus epidermidis and Pseudomonas aeruginosa, by roughly 85% compared with smooth controls [50].

**Evidence in Dentistry:** Lotus-inspired antibiofilm surfaces have shown promising results against oral bacteria. For example, polylactic acid sheets designed to mimic lotus-like surface patterns have been exposed to oral bacteria, including *Streptococcus mutans* and *Lactobacillus* species. Surfaces that were more water-repellent and rougher at the micro- and nano-scales consistently showed less biofilm buildup [51]. This raises interest for possible use on restorations, orthodontic appliances, or prosthetic materials. However, the evidence on translation for dental materials is low, with most of the knowledge coming from in vitro studies on titanium implant coatings. Liquid-infused titanium surfaces (SLIPS, inspired by pitcher plants and lotus leaves) demonstrated sustained anti-adhesive properties against Streptococcus oralis biofilms over 13 days of continuous flow in an oral chamber system, with greater effectiveness at higher flow rates [52].

For dental polymers, one example applied to Polymethyl methacrylate (PMMA) is a superhydrophobic layer made from a fluoropolymer applied to denture base resins, achieving a water contact angle of about 156° and a sliding angle below 1°. This highly water-repellent behavior was linked to reduced bacterial attachment, while the material remained clear and biocompatible. The surface also showed a self-cleaning effect, which could be useful for improving denture hygiene in daily use [53].

Regarding light-cured polymers, a photocurable thiolene elastomer reinforced with nanocellulose and patterned with a lotus-like surface texture has shown strong water repellency, reaching contact angles around 155 degrees, notably higher than those of the smooth version of the same polymer [54]. The formulation itself was not designed for dentistry. Still, it is based on a cross-linked resin composite system, which is conceptually similar to the chemistry used in dental resin composites. In other words, the study shows that lotus-style surface architecture can be integrated into a light-cured composite network, even if it has not yet been adapted to dental resins.

Currently, all the studies are in a very early stage of investigation, mostly proof-of-concept with no validation in clinically relevant preclinical models.

#### 3.2.2. Shark Skin-Inspired Micropatterned Topographies

The dermal denticles of shark skin exhibit aligned micro-riblet structures that reduce drag and prevent microbial accumulation in marine environments [55]. Translating this principle into dental materials, researchers have fabricated microgrooved or ridged surfaces on resin and titanium substrates to inhibit biofilm formation [56]. Studies have shown that patterned resins with submicron-scale grooves disrupt bacterial adhesion by limiting contact area and altering local shear forces [57]. In particular, *S. mutans* showed significantly reduced viability on shark skin–inspired patterns compared to flat controls [58]. These topographies are particularly promising in implant coatings, orthodontic adhesives, and composite restorations, where physical disruption of biofilms is desirable without relying on antimicrobial leachables. However, there is still a need to validate these strategies under intraoral conditions, as wear could change/remove the specific topography in this approach.

#### 3.2.3. Cicada and Dragonfly Wing-Inspired Nanostructures

Possibly the best example of a purely physical bactericidal surface in nature is the wing of the cicada or dragonfly, which is covered in nanopillars or nanospikes. These structures cause mechanical rupture of bacterial membranes upon contact, particularly targeting Gram-negative organisms [59]. In restorative dentistry, this principle has been applied to create nanopatterned titanium and polymer surfaces using techniques such as hydrothermal treatment, nanoimprint lithography, or laser ablation [60,61]. These surfaces demonstrate non-leaching, contact-based killing of oral pathogens, including *Fusobacterium nucleatum* and *Streptococcus gordonii* [62,63,64]. In one adaptation, nanopillared resin coatings fabricated using a soft lithographic mold exhibited a >90% kill rate of *P. gingivalis* within 2 h without compromising the material’s mechanical integrity [65,66]. This approach offers a chemical-free antimicrobial strategy that could be integrated into composite surfaces, aligners, or implant abutments, with reduced concerns about resistance or cytotoxicity [67]. For this strategy, studies are conceptual again. The approach is logical and simple, but the mouth is not. The dental materials in the mouth undergo polishing that alters the surface, in addition to masticatory load and other critical factors that may limit durability and leave the effectiveness in vivo uncertain.

## 4. Integration into Dental Materials

Bioinspired surfaces can be engineered using several micro- and nano-scale engineering techniques compatible with dental materials and processing workflows. Nanoimprint lithography has been successfully applied to resin films and adhesives to generate reproducible micro- and nanopatterns without significantly altering the bulk polymer chemistry or degree of conversion. This can be reached because the nanoimprinting process operates through physical replication mechanisms that do not interfere with the chemical composition or polymerization kinetics of UV-curable resins [66]. Additive manufacturing approaches, including 3D printing with micro-textured molds, offer potential for scalable manufacturing, particularly in CAD/CAM milling blocks, clear aligners, or additive-manufactured composites [68]. However, production must ensure reproducibility of nanostructures, as even minor deviations in height, spacing, or density can significantly reduce bactericidal or antifouling performance.

Laser ablation or femtosecond texturing enables precise generation of microgrooves and nanopillars on titanium or zirconia substrates while preserving mechanical strength [69,70]. Surface etching and hydrothermal treatment have been employed to generate nanopatterning topographies on ceramic and metallic components, producing bactericidal surfaces without the incorporation of leachable antibacterial agents [60,71].

## 5. Biological Validation Under Realistic Conditions

Much of the antibacterial promise attributed to bioinspired dental surfaces rests on evidence generated from overly simplified laboratory models. Most studies still rely on single-species cultures, typically Streptococcus mutans or Pseudomonas aeruginosa, grown under static conditions [71,72]. These systems are easy to standardize, but they do not resemble the biological reality of oral biofilms [72,73]. Inside the mouth, restorations are exposed to cyclic mechanical loading, enzymatic degradation, thermal fluctuations, dynamic pH changes, polymicrobial biofilms, and patient-specific factors [74]. As a result, materials that perform well in laboratory settings often show reduced durability or functionality clinically. For instance, ion-releasing systems may exhibit limited longevity without effective recharge or controlled release mechanisms.

By not exposing the material to ecological competition, metabolic cooperation, spatial structure, and environmental stressors, they tend to inflate apparent antibacterial effects and provide a misleading sense of clinical relevance. This problem is not merely methodological. It reflects a deeper knowledge-translation gap. Findings from controlled in vitro assays represent an early conceptual stage of knowledge, inherently distant from clinical use [75]. As a result, many reported antibacterial benefits remain trapped at the proof-of-concept level, never advancing to technologies that demonstrate meaningful clinical impact (Table 1).

## 6. Challenges in Clinical Translation

Despite significant advances in material design and encouraging laboratory data, the clinical translation of bioinspired, biomimetic, and bioactive restorative materials remains limited. Barriers come from gaps in research or understanding scientific, complex regulatory pathways, and practical clinical implementation challenges that create a “valley of death” between laboratory innovation and patient care [95]. The multifunctional nature of these materials complicates optimization, as enhanced biological activity may compromise mechanical performance. At the same time, the lack of clear definitions and functional classifications for terms such as “bioinspired,” “biomimetic,” and “bioactive” creates confusion in the literature and limits comparability across studies [96]. This lack of clarity also contributes to overstated claims and uncertainty regarding clinical indications.

Biomimetic remineralization systems show promising laboratory outcomes, but it is unclear whether they achieve true intrafibrillar mineralization and long-term functional recovery. Similarly, for antibacterial design, the relative effectiveness of bioinspired surface architectures versus molecular biomimetic approaches under clinically relevant conditions (saliva, polymicrobial biofilm, cycling mastigatory forces, etc.) remains unresolved. Furthermore, current in vitro models may overestimate antimicrobial performance, raising questions about their predictive validity.

Additional barriers relate to manufacturing, scalability, cost, and the lack of long-term clinical evidence [97]. Advanced fabrication techniques, although effective experimentally, are difficult to translate into cost-effective, large-scale production and must remain compatible with existing clinical workflows and handling requirements, important aspects for dentists using these materials in a dental practice. Critically, long-term data remain scarce, with insufficient evidence on durability, surface stability, and degradation under intraoral conditions.

## 7. Conclusions

In summary, bioinspired, biomimetic, and bioactive approaches each play a unique role in shaping the future of restorative dental materials. While bioinspired designs take cues from the structure or properties of natural surfaces, biomimetic strategies go a step further by aiming to reproduce how dental tissues are built and function. Bioactive materials, on the other hand, are created to interact directly with biological systems, often triggering beneficial chemical or cellular responses.

Together, these approaches are opening new possibilities in restorative dentistry. Rather than simply replacing damaged tissue, modern materials are beginning to support regeneration and better integration with the natural oral environment. This shift could lead to restorations that last longer and behave more like real tooth structures.

Looking ahead, there is still a need for deeper research into how these materials perform over time, especially under complex conditions such as those in the mouth. Studies investigating these approaches using clinically relevant models (wear, cycling load, simulated brushing, among others) could improve their feasibility.

Stronger collaboration between researchers and dentists will be important to ensure that promising laboratory findings can be translated into practical treatments. In addition, factors such as durability, safety, and ease of manufacturing must be carefully considered to make these materials viable for dental practice.

## Figures and Tables

**Table 1:** Conceptual classification and maturity of bioinspired, biomimetic, and bioactive dental restorative materials using a knowledge-state framework.

Design Strategy	Primary Design Intent	Dominant Knowledge State *	Intended Outcome	Reference
Bioinspired	Borrow physical or structural motifs from nature	Discovery	- Superhydrophobic resin	[76]
			- Antifouling resin surface	[77]
			- Microgrooved surfaces	[56,58]
			- Nanopillared resin coatings	[66]
Biomimetic	Replicate tissue architecture, gradients, or interfacial behavior	Discovery → Invention	- Remineralization of dentin	[6,32,40,41,42]
			- Remineralization of enamel	[78,79]
			- Oriented HAP crystals to mimic enamel prisms.	[80]
			- Enamel’s compact prism-like structure	[22]
			- Self-healing microcapsules in dental luting cements and resins.	[81,82]
Bioactive	Actively interact with the oral environment through chemical or biological mechanisms	Invention	- Ion-mediated biological responses	[83,84,85]
			- Antibacterial effect	[79,84,86,87,88,89]
			- Promote hydroxyapatite formation	[90]
			- Repel salivary proteins	[89,91]
			- Reactive oxygen species (ROS) generation	[92,93]
			- Selective pH response	[83,94]

* Knowledge states adapted from Lane and Flagg’s discovery–invention–innovation framework, where discovery represents conceptual knowledge, invention reflects embodied and feasible prototypes, and innovation denotes validated, durable solutions adopted in practice [75]. * This is meant as a simple way to think about broader translational trends, not as a rigid or definitive classification.

## Data Availability

No new data were created or analyzed in this study. Data sharing is not applicable to this article.

## Abbreviations

The following abbreviations are used in this manuscript:

CAD/CAM	Computer-Aided Design/Computer-Aided Manufacturing
DEI	Dentin–enamel interface
MMPs	Matrix metalloproteinases
ACP	Amorphous calcium phosphate
MPC	2-methacryloyloxyethyl phosphorylcholine
DMAHDM	Dimethylaminohexadecyl methacrylate
QAM	Quaternary ammonium monomer
AMP	Antimicrobial peptides
ISO	International Organization of Standardization
ROS	Reactive oxygen species

## References

[1] Melo M.A.S., Garcia I.M., Mokeem L., Weir M.D., Xu H.H.K., Montoya C., Orrego S. (2023). Developing Bioactive Dental Resins for Restorative Dentistry. J. Dent. Res..

[2] Kopperud S.E., Tveit A.B., Gaarden T., Sandvik L., Espelid I. (2012). Longevity of posterior dental restorations and reasons for failure. Eur. J. Oral Sci..

[3] Ferracane J.L. (2011). Resin composite—State of the art. Dent. Mater..

[4] Marsh P.D. (2006). Dental plaque as a biofilm and a microbial community—Implications for health and disease. BMC Oral Health.

[5] Marshall G.W., Marshall S.J., Kinney J.H., Balooch M. (1997). The dentin substrate: Structure and properties related to bonding. J. Dent..

[6] Tay F.R., Pashley D.H. (2009). Biomimetic remineralization of resin-bonded acid-etched dentin. J. Dent. Res..

[7] Niu L., Zhang W., Pashley D.H., Breschi L., Mao J., Chen J., Tay F.R. (2014). Biomimetic remineralization of dentin. Dent. Mater..

[8] Zhou Y., Liu K., Zhang H. (2023). Biomimetic Mineralization: From Microscopic to Macroscopic Materials and Their Biomedical Applications. ACS Appl. Bio Mater..

[9] Wang J., Liu Q., Guo Z., Pan H., Liu Z., Tang R. (2023). Progress on Biomimetic Mineralization and Materials for Hard Tissue Regeneration. ACS Biomater. Sci. Eng..

[10] Singer L., Fouda A., Bourauel C. (2023). Biomimetic approaches and materials in restorative and regenerative dentistry: Review article. BMC Oral Health.

[11] Zhou Y., Deng J., Zhang Y., Li C., Wei Z., Shen J., Li J., Wang F., Han B., Chen D. (2022). Engineering DNA-Guided Hydroxyapatite Bulk Materials with High Stiffness and Outstanding Antimicrobial Ability for Dental Inlay Applications. Adv. Mater..

[12] He L.H., Swain M.V. (2008). Understanding the mechanical behaviour of human enamel from its structural and compositional characteristics. J. Mech. Behav. Biomed. Mater..

[13] Beniash E. (2011). Biominerals--hierarchical nanocomposites: The example of bone. Wiley Interdiscip. Rev. Nanomed. Nanobiotechnol..

[14] Varpio L., Paradis E., Uijtdehaage S., Young M. (2020). The Distinctions Between Theory, Theoretical Framework, and Conceptual Framework. Acad. Med..

[15] Melo M.A.S., Garcia I.M., Alluhaidan T., Qaw M., Montoya C., Orrego S., Balhaddad A.A., Mokeem L. (2025). The next frontier in antibacterial dental resins: A 20-year journey of innovation and expectations. Dent. Mater..

[16] Reis A., Feitosa V.P., Chibinski A.C., Favoreto M.W., Gutierrez M.F., Loguercio A.D. (2024). Biomimetic Restorative Dentistry: An evidence-based discussion of common myths. J. Appl. Oral Sci..

[17] Williams D.F. (2022). Biocompatibility pathways and mechanisms for bioactive materials: The bioactivity zone. Bioact. Mater..

[18] Nudelman F., Pieterse K., George A., Bomans P.H.H., Friedrich H., Brylka L.J., Hilbers P.A.J., de With G., Sommerdijk N.A.J.M. (2010). The role of collagen in bone apatite formation in the presence of hydroxyapatite nucleation inhibitors. Nat. Mater..

[19] Parsegian K. (2023). The BMP and FGF pathways reciprocally regulate odontoblast differentiation. Connect. Tissue Res..

[20] Tjäderhane L., Nascimento F.D., Breschi L., Mazzoni A., Tersariol I.L.S., Geraldeli S., Tezvergil-Mutluay A., Carrilho M.R., Carvalho R.M., Tay F.R. (2013). Optimizing dentin bond durability: Control of collagen degradation by matrix metalloproteinases and cysteine cathepsins. Dent. Mater..

[21] Gamea S., Radvar E., Athanasiadou D., Chan R.L., De Sero G., Ware E., Kundi S., Patel A., Horamee S., Hadadi S. (2025). Biomimetic Mineralization of Keratin Scaffolds for Enamel Regeneration. Adv. Healthc. Mater..

[22] Li Y., Ping H., Wei J., Zou Z., Zhang P., Xie J., Jia Y., Xie H., Wang W., Wang K. (2021). Bioprocess-Inspired Room-Temperature Synthesis of Enamel-like Fluorapatite/Polymer Nanocomposites Controlled by Magnesium Ions. ACS Appl. Mater. Interfaces.

[23] Seredin P., Goloshchapov D., Kashkarov V., Emelyanova A., Buylov N., Barkov K., Ippolitov Y., Khmelevskaia T., Mahdy I.A., Mahdy M.A. (2022). Biomimetic Mineralization of Tooth Enamel Using Nanocrystalline Hydroxyapatite under Various Dental Surface Pretreatment Conditions. Biomimetics.

[24] Wong H.M., Zhang Y.Y., Li Q.L. (2022). An enamel-inspired bioactive material with multiscale structure and antibacterial adhesion property. Bioact. Mater..

[25] Oyane A., Sakamaki I., Koga K., Nakamura M., Shitomi K., Miyaji H. (2020). Antibacterial tooth surface created by laser-assisted pseudo-biomineralization in a supersaturated solution. Mater. Sci. Eng. C Mater. Biol. Appl..

[26] Zhang J., Dong H., Liu B., Yang D. (2025). Biomimetic Materials for Antibacterial Applications. Small.

[27] Yu Z., Deng C., Lei T., Wang H., Liu Y., Liu C., Seidi F., Yong Q., Xiao H. (2025). Cationic antibacterial polymers for development of bactericidal materials: Strategies, mechanisms, and applications. Adv. Colloid Interface Sci..

[28] Sharma V.K., Gupta J., Mitra J.B., Srinivasan H., Sakai V.G., Ghosh S.K., Mitra S. (2024). The Physics of Antimicrobial Activity of Ionic Liquids. J. Phys. Chem. Lett..

[29] Tonoyan L., Montagner D., Friel R., O’Flaherty V. (2020). Antimicrobials offered from nature: Peroxidase-catalyzed systems and their mimics. Biochem. Pharmacol..

[30] Huang L., Sun D.-W., Pu H. (2022). Photosensitized Peroxidase Mimicry at the Hierarchical 0D/2D Heterojunction-Like Quasi Metal-Organic Framework Interface for Boosting Biocatalytic Disinfection. Small.

[31] Dong H., Wang D., Deng H., Yin L., Wang X., Yang W., Cai K. (2024). Application of a calcium and phosphorus biomineralization strategy in tooth repair: A systematic review. J. Mater. Chem. B.

[32] Fernández-Romero E., Toledano M., González-Fernández J.F., Osorio R., Vallecillo-Rivas M. (2025). Remineralizing potential of self-assembling peptides on dentinal lesions: A systematic review of the literature. J. Dent..

[33] Yan J., Yang H., Luo T., Hua F., He H. (2022). Application of Amorphous Calcium Phosphate Agents in the Prevention and Treatment of Enamel Demineralization. Front. Bioeng. Biotechnol..

[34] Lei C., Wang K.-Y., Ma Y.-X., Hao D.-X., Zhu Y.-N., Wan Q.-Q., Zhang J.-S., Tay F.R., Mu Z., Niu L.-N. (2024). Biomimetic Self-Maturation Mineralization System for Enamel Repair. Adv. Mater..

[35] Baccolini V., da Silva L.P., Teixeira L., de Sousa R.T., Manarte-Monteiro P. (2025). The Role of Casein Phosphopeptide-Amorphous Calcium Phosphate (CPP-ACP) in White Spot Lesion Remineralization-A Systematic Review. J. Funct. Biomater..

[36] Zhou L., Hou Y.-H., Luo S.-Y., Wang H.-J., Wang H.-R., Guo X.-W., Liu J., Zhang X.-Y., Zhang X. (2026). An amyloid-like protein coating synergizes with a carboxymethyl chitosan/amorphous calcium phosphate nanocomposite for biomimetic dental enamel remineralization. J. Dent..

[37] Iafisco M., Degli Esposti L., Ramírez-Rodríguez G.B., Carella F., Gómez-Morales J., Ionescu A.C., Brambilla E., Tampieri A., Delgado-López J.M. (2018). Fluoride-doped amorphous calcium phosphate nanoparticles as a promising biomimetic material for dental remineralization. Sci. Rep..

[38] Carrilho M.R.D.O., Tay F.R., Pashley D.H., Tjäderhane L., Marins Carvalho R. (2005). Mechanical stability of resin–dentin bond components. Dent. Mater..

[39] Liang S., Gao X., Li X., Yao C., Huang C. (2025). Promotion of Biomimetic Mineralization via Preinfiltration of Mineral Precursors. ACS Appl. Mater. Interfaces.

[40] Palmer L.C., Newcomb C.J., Kaltz S.R., Spoerke E.D., Stupp S.I. (2008). Biomimetic systems for hydroxyapatite mineralization inspired by bone and enamel. Chem. Rev..

[41] Yucesoy D.T., Fong H., Hamann J., Hall E., Dogan S., Sarikaya M. (2023). Biomimetic Dentin Repair: Amelogenin-Derived Peptide Guides Occlusion and Peritubular Mineralization of Human Teeth. ACS Biomater. Sci. Eng..

[42] Yang S.H., Tian Z.L., Wang H.M., Sun D., Qiao S.W., Shi Z.S., He X., Zhu S. (2025). Multifunctional Primer for Dentin Bonding via Biomimetic Mineralization. J. Dent. Res..

[43] Quero I.B., Dias P.C., Magalhães N.L., Faraoni J.J., Palma-Dibb R.G. (2025). Biomodification of coronal bovine dentin with chitosan solutions associated with modified nano-hydroxyapatite and Biosilicate^®^. Dent. Mater..

[44] Wu L., Cao X., Meng Y., Huang T., Zhu C., Pei D., Weir M.D., Oates T.W., Lu Y., Xu H.H.K. (2022). Novel bioactive adhesive containing dimethylaminohexadecyl methacrylate and calcium phosphate nanoparticles to inhibit metalloproteinases and nanoleakage with three months of aging in artificial saliva. Dent. Mater..

[45] Bhushan B., Jung Y.C. (2011). Natural and biomimetic artificial surfaces for superhydrophobicity, self-cleaning, low adhesion, and drag reduction. Prog. Mater. Sci..

[46] Zhang J., Chen Y., Du S., Du M., Jeong H.E., Jiang R., Zhao J., Ren L. (2025). Addressing bacterial threats in a post-antibiotic era: Bioinspired strategies for antibacterial surface design. Adv. Bionics.

[47] Saubade F., Pilkington L.I., Liauw C.M., Gomes L.C., McClements J., Peeters M., El Mohtadi M., Mergulhão F.J., Whitehead K.A. (2021). Principal Component Analysis to Determine the Surface Properties That Influence the Self-Cleaning Action of Hydrophobic Plant Leaves. Langmuir.

[48] Nosonovsky M., Bhushan B. (2009). Superhydrophobic surfaces and emerging applications: Non-adhesion, energy, green engineering. Curr. Opin. Colloid Interface Sci..

[49] Yuan Y., Hays M.P., Hardwidge P.R., Kim J. (2017). Surface characteristics influencing bacterial adhesion to polymeric substrates. RSC Adv..

[50] Cao Y., Jana S., Bowen L., Tan X., Liu H., Rostami N., Brown J., Jakubovics N.S., Chen J. (2019). Hierarchical Rose Petal Surfaces Delay the Early-Stage Bacterial Biofilm Growth. Langmuir.

[51] Venkatachalam G., Venkatesan N., Vatsal S., Chavan I., Bakshi A., Doble M. (2025). Biofilm-Forming Ability of Infectious Organisms on Biomimetic Surfaces An In Vitro and Machine-Learning Analysis. ACS Omega.

[52] Doll K., Yang I., Fadeeva E., Kommerein N., Szafrański S.P., Bei der Wieden G., Greuling A., Winkel A., Chichkov B.N., Stumpp N.S. (2019). Liquid-Infused Structured Titanium Surfaces: Antiadhesive Mechanism to Repel Streptococcus oralis Biofilms. ACS Appl. Mater. Interfaces.

[53] Cheng Q., Cao D., Liu X., Zheng Y., Shi Z., Zhu S., Cui Z. (2019). Superhydrophobic coatings with self-cleaning and antibacterial adhesion properties for denture base. J. Mech. Behav. Biomed. Mater..

[54] Balkan A., Sola E., Karasu F., Leterrier Y. (2024). Photocurable Thiol–Ene/Nanocellulose Elastomeric Composites for Bioinspired and Fluorine-Free Superhydrophobic Surfaces. ACS Appl. Mater. Interfaces.

[55] Schumacher J.F., Carman M.L., Estes T.G., Feinberg A.W., Wilson L.H., Callow M.E., Callow J.A., Finlay J.A., Brennan A.B. (2007). Engineered antifouling microtopographies—Effect of feature size, geometry, and roughness on settlement of zoospores of the green alga Ulva. Biofouling.

[56] Mei S., Wang H., Wang W., Tong L., Pan H., Ruan C., Ma Q., Liu M., Yang H., Zhang L. (2014). Antibacterial effects and biocompatibility of titanium surfaces with graded silver incorporation in titania nanotubes. Biomaterials.

[57] Kreve S., Dos Reis A.C. (2022). Effect of surface properties of ceramic materials on bacterial adhesion: A systematic review. J. Esthet. Restor. Dent..

[58] Arango-Santander S., Gonzalez C., Aguilar A., Cano A., Castro S., Sanchez-Garzon J., Franco J. (2020). Assessment of Streptococcus Mutans Adhesion to the Surface of Biomimetically-Modified Orthodontic Archwires. Coatings.

[59] Hasan J., Webb H.K., Truong V.K., Pogodin S., Baulin V.A., Watson G.S., Watson J.A., Crawford R.J., Ivanova E.P. (2013). Selective bactericidal activity of nanopatterned superhydrophobic cicada Psaltoda claripennis wing surfaces. Appl. Microbiol. Biotechnol..

[60] Truong V.K., Lapovok R., Estrin Y.S., Rundell S., Wang J.Y., Fluke C.J., Crawford R.J., Ivanova E.P. (2010). The influence of nano-scale surface roughness on bacterial adhesion to ultrafine-grained titanium. Biomaterials.

[61] Hasan J., Chatterjee K. (2015). Recent advances in engineering topography mediated antibacterial surfaces. Nanoscale.

[62] Hasan J., Jain S., Padmarajan R., Purighalla S., Sambandamurthy V.K., Chatterjee K. (2018). Multi-scale surface topography to minimize adherence and viability of nosocomial drug-resistant bacteria. Mater. Des..

[63] Montoya C., Roldan L., Yu M., Valliani S., Ta C., Yang M., Orrego S. (2023). Smart dental materials for antimicrobial applications. Bioact. Mater..

[64] Choi S., Jo Y.-H., Luke Yeo I.-S., Yoon H.-I., Lee J.-H., Han J.-S. (2023). The effect of surface material, roughness and wettability on the adhesion and proliferation of Streptococcus gordonii, Fusobacterium nucleatum and Porphyromonas gingivalis. J. Dent. Sci..

[65] Modaresifar K., Azizian S., Ganjian M., Fratila-Apachitei L.E., Zadpoor A.A. (2019). Bactericidal effects of nanopatterns: A systematic review. Acta Biomater..

[66] Xu L.-C., Siedlecki C.A. (2012). Submicron-textured biomaterial surface reduces staphylococcal bacterial adhesion and biofilm formation. Acta Biomater..

[67] Priya S., Malviya R., Srivastava S., Siang T.C., Aseeri A.A. (2026). Bioinspired nanostructured surfaces for antimicrobial and antifouling applications. Colloid Interface Sci. Commun..

[68] Oros D., Penčić M., Orošnjak M., Kedziora S. (2025). Additive Manufacturing Technologies and Their Applications in Dentistry: A Systematic Literature Review. Appl. Sci..

[69] Wu X., Ao H., He Z., Wang Q., Peng Z. (2022). Surface Modification of Titanium by Femtosecond Laser in Reducing Bacterial Colonization. Coatings.

[70] Cunha A., Elie A.-M., Plawinski L., Serro A.P., Botelho Do Rego A.M., Almeida A., Urdaci M.C., Durrieu M.-C., Vilar R. (2016). Femtosecond laser surface texturing of titanium as a method to reduce the adhesion of Staphylococcus aureus and biofilm formation. Appl. Surf. Sci..

[71] Wu S., Altenried S., Zogg A., Zuber F., Maniura-Weber K., Ren Q. (2018). Role of the Surface Nanoscale Roughness of Stainless Steel on Bacterial Adhesion and Microcolony Formation. ACS Omega.

[72] Ibrahim M.S., Garcia I.M., Kensara A., Balhaddad A.A., Collares F.M., Williams M.A., Ibrahim A.S., Lin N.J., Weir M.D., Xu H.H.K. (2020). How we are assessing the developing antibacterial resin-based dental materials? A scoping review. J. Dent..

[73] Ramachandra S.S., Wright P., Han P., Abdal-hay A., Lee R.S.B., Ivanovski S. (2023). Evaluating models and assessment techniques for understanding oral biofilm complexity. MicrobiologyOpen.

[74] Alluhaidan T., Qaw M., Garcia I.M., Montoya C., Orrego S., Melo M.A. (2024). Seeking Endurance: Designing Smart Dental Composites for Tooth Restoration. Designs.

[75] Lane J.P., Flagg J.L. (2010). Translating three states of knowledge--discovery, invention, and innovation. Implement. Sci..

[76] Zhang S., He X., Liu F., Huang X., Mai S., He J. (2025). Preparation of dental resin composites with antibacterial adhesion against Streptococcus mutans using fluorinated and silicon containing dimethacrylates. Dent. Mater..

[77] Zhang N., Ma J., Melo M.A.S., Weir M.D., Bai Y., Xu H.H.K. (2015). Protein-repellent and antibacterial dental composite to inhibit biofilms and caries. J. Dent..

[78] Cheng L., Zhang K., Melo M.A.S., Weir M.D., Zhou X., Xu H.H.K. (2012). Anti-biofilm dentin primer with quaternary ammonium and silver nanoparticles. J. Dent. Res..

[79] Farjaminejad R., Farjaminejad S., Garcia-Godoy F., Jalali M. (2025). The Role of Bioactive Glasses in Caries Prevention and Enamel Remineralization. Appl. Sci..

[80] Chen Z., Miao Z., Zhang P., Xiao H., Liu H., Ding C., Tan H., Li J. (2019). Bioinspired enamel-like oriented minerals on general surfaces: Towards improved mechanical properties. J. Mater. Chem. B.

[81] Wu J., Zhang Q., Weir M.D., Oates T.W., Zhou C., Chang X., Xu H.H.K. (2017). Novel self-healing dental luting cements with microcapsules for indirect restorations. J. Dent..

[82] Wu J., Weir M.D., Zhang Q., Zhou C., Melo M.A.S., Xu H.H.K. (2016). Novel self-healing dental resin with microcapsules of polymerizable triethylene glycol dimethacrylate and N,N-dihydroxyethyl-p-toluidine. Dent. Mater..

[83] Zhang L., Weir M.D., Chow L.C., Antonucci J.M., Chen J., Xu H.H.K. (2016). Novel rechargeable calcium phosphate dental nanocomposite. Dent. Mater..

[84] Melo M.A.S., Cheng L., Weir M.D., Hsia R.-C., Rodrigues L.K.A., Xu H.H.K. (2013). Novel dental adhesive containing antibacterial agents and calcium phosphate nanoparticles. J. Biomed. Mater. Res. B Appl. Biomater..

[85] Cai J.-N., Choi H.-M., Song K.-Y., Jeon J.-G. (2022). The reciprocal interaction between fluoride release of glass ionomers and acid production of Streptococcus mutans biofilm. J. Oral Microbiol..

[86] Degli Esposti L., Ionescu A.C., Carella F., Adamiano A., Brambilla E., Iafisco M. (2022). Antimicrobial Activity of Remineralizing Ion-Doped Amorphous Calcium Phosphates for Preventive Dentistry. Front. Mater..

[87] Zhou W., Chen H., Weir M.D., Oates T.W., Zhou X., Wang S., Cheng L., Xu H.H.K. (2023). Novel bioactive dental restorations to inhibit secondary caries in enamel and dentin under oral biofilms. J. Dent..

[88] Li F., Weir M.D., Chen J., Xu H.H.K. (2013). Comparison of quaternary ammonium-containing with nano-silver-containing adhesive in antibacterial properties and cytotoxicity. Dent. Mater..

[89] Zhang N., Zhang K., Melo M.A.S., Weir M.D., Xu D.J., Bai Y., Xu H.H.K. (2017). Effects of Long-Term Water-Aging on Novel Anti-Biofilm and Protein-Repellent Dental Composite. Int. J. Mol. Sci..

[90] Tezvergil-Mutluay A., Seseogullari-Dirihan R., Feitosa V.P., Cama G., Brauer D.S., Sauro S. (2017). Effects of Composites Containing Bioactive Glasses on Demineralized Dentin. J. Dent. Res..

[91] Cao L., Wu J., Zhang Q., Baras B., Bhadila G., Li Y., Melo M.A.S., Weir M.D., Bai Y., Zhang N. (2019). Novel Protein-Repellent and Antibacterial Resins and Cements to Inhibit Lesions and Protect Teeth. Int. J. Polym. Sci..

[92] Wang R., Jia C., Zheng N., Liu S., Qi Z., Wang R., Zhang L., Niu Y., Pan S. (2023). Effects of photodynamic therapy on Streptococcus mutans and enamel remineralization of multifunctional TiO2-HAP composite nanomaterials. Photodiagn. Photodyn. Ther..

[93] Gao L., Zhuang J., Nie L., Zhang J., Zhang Y., Gu N., Wang T., Feng J., Yang D., Perrett S. (2007). Intrinsic peroxidase-like activity of ferromagnetic nanoparticles. Nat. Nanotech..

[94] Cheng L., Zhang K., Zhou C.-C., Weir M.D., Zhou X.-D., Xu H.H.K. (2016). One-year water-ageing of calcium phosphate composite containing nano-silver and quaternary ammonium to inhibit biofilms. Int. J. Oral Sci..

[95] Wang L., Guo X., Chen J., Zhen Z., Cao B., Wan W., Dou Y., Pan H., Xu F., Zhang Z. (2022). Key considerations on the development of biodegradable biomaterials for clinical translation of medical devices: With cartilage repair products as an example. Bioact. Mater..

[96] Ferracane J.L., Sidhu S.K., Melo M.A.S., Yeo I.-S.L., Diogenes A., Darvell B.W. (2023). Bioactive dental materials: Developing, promising, confusing. JADA Found. Sci..

[97] Frisch E., Clavier L., Belhamdi A., Vrana N.E., Lavalle P., Frisch B., Heurtault B., Gribova V. (2023). Preclinical in vitro evaluation of implantable materials: Conventional approaches, new models and future directions. Front. Bioeng. Biotechnol..

